# Supplementary material for: Role and mechanism of NCAPD3 in promoting malignant behaviors in gastric cancer
Source: Front Pharmacol. 2024 Apr 22;15:1341039. doi: 10.3389/fphar.2024.1341039 (PMC11070777; doi:10.3389/fphar.2024.1341039)
Supplement: Supplementary file 11 [file DataSheet2.ZIP › GSEA/Oncogenic signature/my_analysis.Gsea.1599461962066/MEK_UP.V1_UP.html]

Details for gene set MEK\_UP.V1\_UP[GSEA]

|  || Dataset | filtered\_dataset.sample\_info.cls#WT\_versus\_NCAPD3\_MUT |
| Phenotype | sample\_info.cls#WT\_versus\_NCAPD3\_MUT |
| Upregulated in class | NCAPD3\_MUT |
| GeneSet | MEK\_UP.V1\_UP |
| Enrichment Score (ES) | -0.4175513 |
| Normalized Enrichment Score (NES) | -2.2743065 |
| Nominal p-value | 0.002538071 |
| FDR q-value | 0.008003171 |
| FWER p-Value | 0.018 |
Table: GSEA Results Summary

  

Fig 1: Enrichment plot: MEK\_UP.V1\_UP      
 Profile of the Running ES Score & Positions of GeneSet Members on the Rank Ordered List

  

| SYMBOL | TITLE | RANK IN GENE LIST | RANK METRIC SCORE | RUNNING ES | CORE ENRICHMENT || 1 | 79789 | CLMN | 160 | 0.717 | -0.0730 | No |
| 2 | 64856 | VWA1 | 259 | 0.623 | -0.1067 | No |
| 3 | 2244 | FGB | 321 | 0.582 | -0.1160 | No |
| 4 | 81031 | SLC2A10 | 539 | 0.453 | -0.2462 | No |
| 5 | 1119 | CHKA | 670 | 0.390 | -0.3171 | No |
| 6 | 25825 | BACE2 | 756 | 0.344 | -0.3581 | No |
| 7 | 6558 | SLC12A2 | 839 | 0.276 | -0.4010 | Yes |
| 8 | 360 | AQP3 | 857 | -0.263 | -0.3975 | Yes |
| 9 | 55450 | CAMK2N1 | 861 | -0.269 | -0.3836 | Yes |
| 10 | 2150 | F2RL1 | 887 | -0.304 | -0.3835 | Yes |
| 11 | 3691 | ITGB4 | 926 | -0.352 | -0.3899 | Yes |
| 12 | 4680 | CEACAM6 | 955 | -0.374 | -0.3878 | Yes |
| 13 | 10123 | ARL4C | 957 | -0.377 | -0.3659 | Yes |
| 14 | 9314 | KLF4 | 972 | -0.389 | -0.3527 | Yes |
| 15 | 6286 | S100P | 1011 | -0.412 | -0.3556 | Yes |
| 16 | 11138 | TBC1D8 | 1026 | -0.424 | -0.3403 | Yes |
| 17 | 1543 | CYP1A1 | 1058 | -0.451 | -0.3358 | Yes |
| 18 | 4293 | MAP3K9 | 1116 | -0.492 | -0.3476 | Yes |
| 19 | 5097 | PCDH1 | 1132 | -0.502 | -0.3284 | Yes |
| 20 | 3887 | KRT81 | 1168 | -0.536 | -0.3216 | Yes |
| 21 | 2119 | ETV5 | 1176 | -0.545 | -0.2939 | Yes |
| 22 | 6310 | ATXN1 | 1177 | -0.547 | -0.2611 | Yes |
| 23 | 64759 | TNS3 | 1197 | -0.572 | -0.2406 | Yes |
| 24 | 23657 | SLC7A11 | 1205 | -0.581 | -0.2108 | Yes |
| 25 | 8061 | FOSL1 | 1222 | -0.597 | -0.1866 | Yes |
| 26 | 54491 | FAM105A | 1241 | -0.619 | -0.1625 | Yes |
| 27 | 23508 | TTC9 | 1271 | -0.660 | -0.1439 | Yes |
| 28 | 80328 | ULBP2 | 1277 | -0.664 | -0.1077 | Yes |
| 29 | 4739 | NEDD9 | 1322 | -0.741 | -0.0952 | Yes |
| 30 | 2650 | GCNT1 | 1342 | -0.781 | -0.0621 | Yes |
| 31 | 22936 | ELL2 | 1370 | -0.882 | -0.0288 | Yes |
| 32 | 23650 | TRIM29 | 1384 | -0.951 | 0.0189 | Yes |
Table: GSEA details [plain text format]

  

Fig 2: MEK\_UP.V1\_UP      
 Blue-Pink O' Gram in the Space of the Analyzed GeneSet

  

Fig 3: MEK\_UP.V1\_UP: Random ES distribution      
 Gene set null distribution of ES for **MEK\_UP.V1\_UP**
